# Validation of New Methods of Using Simulated Whole-Body Movements as Implicit Indicators of Sound and Odor Preferences

**DOI:** 10.3389/fpsyg.2021.659269

**Published:** 2021-08-04

**Authors:** Donato Cereghetti, Pauline Faye, Laetitia Gros, Lucas Mahé, Emmanuelle Diaz, Isabelle Cayeux, Théo Heritier, Rémy Versace

**Affiliations:** ^1^Firmenich SA, Geneva, Switzerland; ^2^Le Sensolier, Paris, France; ^3^Orange Labs, Lannion, France; ^4^EMC Laboratory, Institute of Psychology, Lyon2 University, Lyon, France; ^5^PSA Groupe, Vélizy-Villacoublay, France; ^6^Silliker SAS, Mérieux NutriSciences, Cergy-Pontoise, France

**Keywords:** consumer, preferences, motivation, approach/avoidance, implicit measures, sounds, odors

## Abstract

Would you get close to a stinky perfume bottle or to a loudspeaker producing noise? In this paper, we present two procedures that allowed us to assess the ability of auditory and olfactory cues to elicit automatic approach/avoidance reactions toward their sources. The procedures resulted from an adaptation of the Visual Approach/Avoidance by the Self Task (VAAST; Rougier et al., 2018), a task having the peculiarity of simulating approach/avoidance reactions by using visual feedback coming from the whole-body movements. In the auditory VAAST (Experiment 1), participants were instructed to move forward or backward from a loudspeaker that produced spoken words differentiated by their level of distortion and thus by their hedonic value. In the olfactory VAAST (Experiment 2), participants were asked to move forward or backward from a perfume bottle that delivered pleasant and unpleasant odors. We expected, consistent with the approach/avoidance compatibility effect, shorter latencies for approaching positive stimuli and avoiding negative stimuli. In both experiments, we found an effect of the quality of the emotional stimulus on forward actions of participants, with undistorted words and pleasant odors inducing faster forward movements compared with that for distorted words and unpleasant odors. Notably, our results further suggest that the VAAST can successfully be used with implicit instructions, i.e., without requiring participants to explicitly process the valence of the emotional stimulus (in Experiment 1) or even the emotional stimulus itself (in Experiment 2). The sensitivity of our procedures is analyzed and its potential in cross-modal and (contextualized) consumer research discussed.

## Introduction

How can the hedonic value of a product be measured alone, without being simultaneously contaminated by something else? Most often, consumer judgments are collected by using direct measures (e.g., rating scales, questionnaires, or semi-directive interviews). Although such methods proved their ability to discriminate products, they are also subject to several biases. First, consumer judgments are likely to be affected by a social desirability bias (Edwards, 1953) and may rely on individual introspective skills (Nisbett and Wilson, 1977). In addition, since the expression of a judgment requires the translation of a perceptual feeling into an explicit response, it is difficult to dissociate, in consumer judgment, the variability due to the perceptual feeling from the variability due to its explicit translation. Finally, many biases related to the way individuals use scales when quantifying their judgment have been reported, among which we mention contraction biases, centering biases, and logarithmic biases (for a review, see Poulton, 1989). To avoid such biases, it is necessary to use indirect measures that are likely to reflect the hedonic value of the product.

The hedonic value refers to the pleasure provided by the confrontation with a stimulus; it is therefore intrinsically linked to the emotions elicited in the individual by the stimulus. Etymologically, the term “emotion” refers to a setting in motion, a change from an initial state. Such changes are manifested at different levels: at the physiological level (brain electrophysiological responses, cardiovascular, respiratory, skin conductance responses, etc.) and at the behavioral level (motor reactions, approach/avoidance reactions, etc.). It is for this reason that many studies have used these changes as implicit indicators of the emotion felt; the underlying idea is that the more intense the emotion, the more important these changes must be.

From an adaptive point of view, it is indeed obvious that one of the primary and most important behavioral responses toward a stimulus is whether it should be approached or avoided. Approach/avoidance procedures were developed to investigate this primary behavioral response; the basic assumption is that positive stimuli elicit approach reactions, whereas negative stimuli elicit avoidance reactions. Approach/avoidance tendencies are typically explored in tasks that require arm and/or hand movements in response to emotional stimuli. In these tasks, reaction times (RTs) are usually recorded, and participants are expected to produce faster responses to approach positive stimuli and to avoid negative stimuli compared with the reverse, which is known as the approach/avoidance compatibility effect. Approach/avoidance tendencies have been investigated by using a large range of different apparatuses and procedures, including the lever or joystick task (e.g., Chen and Bargh, 1999), the modified keyboard task (e.g., Alexopoulos and Ric, 2007), the button stand task (e.g., Rotteveel and Phaf, 2004), and the zoom-feedback joystick task (e.g., Rinck and Becker, 2007). Approach/avoidance tendencies have, moreover, been explored by using different types of stimuli, including words (e.g., Solarz, 1960; Markman and Brendl, 2005; Alexopoulos and Ric, 2007), pseudo-words (e.g., Carr et al., 2016), pictures (e.g., Rinck and Becker, 2007; Saraiva et al., 2013), and faces (e.g., Paulus and Wentura, 2016).

The approach/avoidance compatibility effect was first identified in a study performed by Chen and Bargh (1999), in which participants were required to perform arm flexions and extensions by pulling/pushing a lever in response to emotional words. Their results showed that participants provided faster responses to positive words when pulling a lever toward them and to negative words when pushing the lever away (as compared with the reverse; Experiment 1) and that this occurred even when the task did not require participants to explicitly evaluate the valence of the emotional stimulus (Experiment 2). From these findings, Chen and Bargh (1999) postulated the existence of an automatic, unconscious link between the stimulus evaluation and specific motor responses, with positive stimuli activating arm flexion (approach reaction; i.e., move the stimuli toward the self) and negative stimuli activating arm extension (avoidance reaction; i.e., move the stimuli away from the self). Although a number of studies identified the compatibility effect described in Chen and Bargh (1999), others failed to reproduce it or found the opposite effect, challenging *de facto* the hypothesis that a direct, hard-wired link between evaluation and behavior may exist (Wentura et al., 2000; Markman and Brendl, 2005; Lavender and Hommel, 2007; Rinck and Becker, 2007; Eder and Rothermund, 2008; Paladino and Castelli, 2008; Seibt et al., 2008; Krieglmeyer et al., 2010; Rotteveel et al., 2015). Indeed, arm flexion can be interpreted either as an approach (bringing a positive stimulus, e.g., a pizza, closer to the self) or as avoidance (withdrawing the hand from a negative stimulus, e.g., a spider), and arm extension can be interpreted either as approach (reaching a positive stimulus with the hand, e.g., a pizza) or as avoidance (pushing a negative stimulus, e.g., a spider, away from the self); this depends on contextual factors, on individual's goals (e.g., Bamford and Ward, 2008), and on the frame of reference (self-related vs. object-related; e.g., Seibt et al., 2008).

Not only are results across studies relatively inconsistent, but the compatibility effect sizes reported in the literature are also generally low to moderate (Phaf et al., 2014), suggesting the need to develop new procedures. In that spirit, and based on an embodied approach to cognition (e.g., Niedenthal, 2007; van Dantzig et al., 2009; Versace et al., 2014), Rougier et al. (2018) suggested that a sensorimotor task that simulates the visual information coming from the whole-body movements should produce stronger and more replicable effects. They then developed a new procedure, the Visual Approach/Avoidance by the Self Task (VAAST). This procedure no longer relies on sensorimotor indices limited to arm and hand movements, but rather on sensorimotor indices provided by a realistic visual flow that simulates the whole-body movements in relation to the emotional stimulus. Rougier et al. (2018) evaluated the sensitivity of this measure in a series of six experiments, in which participants had to approach or avoid emotional words as quickly and accurately as possible by using a button box. Notably, in each trial, participants were required to press the corresponding response key four times consecutively in order to complete a single forward or backward movement. After each key press, the whole visual scene (i.e., the surrounding environment and the target word) was zoomed in or out by 10%, giving the visual impression of walking forward or backward to the target as a consequence of their response. In their experiments, Rougier et al. (2018) showed that the VAAST can produce large and replicable compatibility effects.

Despite these promising results, the VAAST should be considered a relatively new procedure, and there are many opportunities for further developments and improvements. We identified three of them. First, previous research has focused only on the visual domain. We believe that this procedure has great potential for cross-modal research and thus deserve to be further investigated by considering other sensory modalities. Second, in the original VAAST (Rougier et al., 2018), participants were asked to move forward or backward from words, which should be considered a non-ecological task. We believe that the VAAST procedure has the potential to be further applied in more (simulated) ecological contexts by using virtual 3D objects located in the visual scene as target stimuli instead of words. Third, although the VAAST has been largely validated by using explicit instructions, the use of implicit instructions still remains underinvestigated. Indeed, in five of the six experiments presented in Rougier et al. (2018), the task required participants to explicitly process the valence of the emotional stimuli (i.e., participants were asked to categorize words as being positive or negative by pressing two response keys). Only in Experiment 5 were participants not required to attentively attend to the stimulus valence. In this experiment, participants were asked to determine whether a sequence of letters (e.g., “nlkjdsOaq”) contained a capital letter or not, while the emotional words were primed for a very short duration (30 ms) with a pre-mask (the sequence “WXWXWXWXW” displayed for 50 ms) and a post-mask (the sequence “@W@W@W@W” displayed for 50 ms).

The purpose of our study was therefore threefold. First, we aimed to extend the VAAST to other sensory modalities by using spoken words (section Experiment 1) and odors (section Experiment 2) as emotional stimuli. That is, we aimed to measure the ability of auditory and olfactory cues to elicit automatic reactions toward their source (i.e., a loudspeaker in Experiment 1 and a perfume bottle in Experiment 2). Notably, the sources were situated in a congruent surrounding environment (i.e., a living room in Experiment 1 and a bathroom in Experiment 2), thus ensuring the ecological validity of the task. Note that in both studies, participants were explicitly informed that auditory and olfactory cues “came from” the loudspeaker and from the perfume bottle, respectively, thus reinforcing the link between the cues and their source. Second, we aimed to provide further evidence concerning the ability of VAAST to measure approach/avoidance reactions for stimuli that differ in their hedonic value. In Experiment 1, spoken words were presented at different levels of distortion (i.e., undistorted, slightly distorted, and moderately distorted); we assume that the more distorted the signal, the more unpleasant the stimulus. In Experiment 2, we used odors that strongly differ in their liking value. We expected, consistent with the approach/avoidance compatibility effect, positive cues to promote an approach reaction and negative cues to promote an avoidance reaction. That is, we expected faster RTs in approaching positive stimuli and avoiding negative stimuli than the reverse. Third, we aimed to show that approach/avoidance compatibility effects can be observed without requiring participants to explicitly process the valence of the stimuli, even, in Experiment 2, without having to explicitly process the stimuli that induce emotions. In Experiment 1, participants had to indicate whether the spoken word represented a living being or an inanimate object, regardless of the level of distortion. In Experiment 2, participants had to judge whatever the perfume bottle was bent toward to the right or to the left; thus, they no longer had to explicitly deal with the stimuli that induce emotions.

## Experiment 1

### Materials and Methods

#### Participants

Twenty-six students from the University of Lyon (13 females, 11 males, two no answers) voluntarily participated for course credits. The average age of the panel was 21.8 years (*SD* = 3.0). The experiment was conducted in accordance with the ethical principles stated in the Declaration of Helsinki. All participants provided written informed consent and reported having a normal or corrected vision and normal hearing. A participant was excluded because of technical issues. The analysis was thus performed on the remaining 25 participants. To the best of our knowledge, this is the first study aiming to adapt the VAAST to auditory stimuli. A priori determination of sample size was therefore not possible. Consequently, we conducted a sensitivity power analysis using G^*^Power 3 (Faul et al., 2007) to identify the smallest effect size that our sample would be able to detect in paired *t*-tests performed in RT analysis (section A-VAAST: Reaction Times). The analysis revealed that our sample size (*n* = 25) could reliably detect the effect sizes of Cohen's *d*_*z*_ = 0.51 (one-tailed), assuming α = 0.05 and power (*1 –* β) = 0.80.

#### Materials

##### Visual Scene

**Figure 1:**
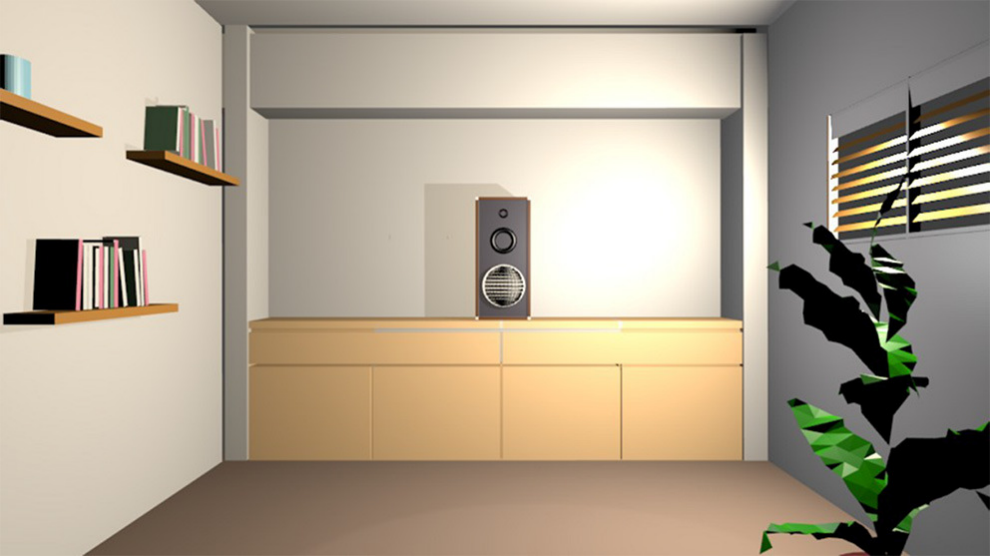
Visual scene of the Auditory Visual Approach/Avoidance by the Self Task (A-VAAST).

A virtual visual scene was designed for the Auditory VAAST (A-VAAST) with 3D Blender. The visual scene consisted of a living room presented in the first-person view (Figure 1). A loudspeaker (i.e., the source of auditory cues) was placed in the center of the visual scene, just above a media cabinet.

##### Auditory Stimuli

Ten neutral words were used in the A-VAAST. Five words represented living beings (“pélican,” “renard,” “cycliste,” “tortue,” “fourmi”; i.e., “pelican,” “fox,” “cyclist,” “turtle,” “ant”) and the other five words represented inanimate objects (“tabouret,” “bouée,” “marmite,” “volant,” “cloche”; i.e., “stool,” “buoy,” “stockpot,” “steering wheel,” “bell”). The words were selected on the basis of a published normative study (Bonin et al., 2003) where participants (*n* = 97) were asked to evaluate the concreteness, imageability, subjective frequency, and emotional valence of 866 French words with a 5-point scale. In particular, we selected ten neutral (valence: *M* = 3.1, *SD* = 0.1) and medium-frequent words (frequency: *M* = 2.7, *SD* = 0.6) while controlling the number of syllables across the two-word categories. In addition, each word started with a different phoneme to avoid the implicit statistical learning of participants. The words uttered by a female speaker were recorded with a Zoom H2 high-definition microphone and normalized in level with an active speech level equalization at −26 dBov, using the implementation of the ITU-T Recommendation P.56 (2011) given in the ITU-T Software Tool Library (ITU-T Recommendation G.191, 2019; see program “sv56demo”). Spoken words were then degraded in quality with noise by using the modulated noise reference unit (ITU-T Recommendation P810, 1996) at a level Q of 22 (slightly distorted) and 15 dB (moderately distorted), with Q being the ratio of speech power to modulated noise power.

#### Procedure

Participants were seated on a chair in front of a computer screen (with a refresh rate of 60 Hz) and were wearing headphones (Sennheiser HD206). Each participant's head position was fixed with the aid of a chin rest such that the distance between the eyes and the screen was 50 cm. Participants were told that they would be immersed in a virtual environment, i.e., a living room equipped with a loudspeaker emitting spoken words. Participants were then instructed to move forward or backward from the loudspeaker as quickly and accurately as possible, depending on the category of the spoken word (i.e., living being, inanimate object). The correspondence between word category and the approach/avoidance action was counterbalanced across participants: Half of the participants had to approach the loudspeaker when the word belonged to the living being category and avoid it when the word belonged to the inanimate object category, whereas the remaining half had to approach the loudspeaker when the word belonged to the inanimate object category and avoid it when the word belonged to the living being category.

At beginning of each trial, the visual scene was presented full screen and participants had to press the “start” key (key 5 of the numeric keypad) to start the trial. Five hundred milliseconds later, a fixation point appeared in the center of the loudspeaker for 500 ms, after which a spoken word was presented on the headphones at a given level of sound distortion (undistorted, slightly distorted, or moderately distorted). Depending on the spoken word category, participants had to press key 8 of the numeric keypad three times consecutively to approach the loudspeaker and key 2 of the numeric keypad to move away from it. After each correct key-press response, the whole visual scene was zoomed in or out by 10%, giving the visual impression of walking forward or backward as a consequence of the approach/avoidance action of participants (Rougier et al., 2018). Following an incorrect response, a red capital “X” was presented for 100 ms, together with an error beep sound, and participants were required to provide the correct response to trigger the visual flow. The trial terminated after three key presses in the same direction (i.e., a complete forward or backward movement) and was followed by a black background that masked the visual scene for 1,000 ms, after which the next trial started. Although participants had to press the relevant key three times consecutively to approach or avoid the loudspeaker, we considered accuracy and RT only for the first action of the participants (see Rougier et al., 2018).

Designed with OpenSesame (Mathôt et al., 2012), the A-VAAST consisted of two phases, namely, practice and test. The training phase was composed of 30 trials in which each word was presented in a randomized order at each level of distortion (i.e., undistorted, slightly distorted, and moderately distorted). In the testing phase, the same 30-item cycle was repeated four times, resulting in a total of 120 trials presented in a randomized order. Participants were thus exposed to 20 trials per each experimental condition level (action × sound quality), thus resulting in 60 “move forward” and 60 “move backward” trials.

### Results

Experimental data and analysis scripts are available on the OpenScience Framework platform at https://osf.io/jkr7m/

#### A-VAAST: Accuracy

Accuracy of the participants on the A-VAAST across the six conditions is summarized in Table 1. We performed a repeated-measures ANOVA with the factors sound quality (undistorted, slightly distorted, and moderately distorted) and action (“move forward,” “move backward”) as within-subject factors for the response accuracy of participants. According to the results, accuracy was not influenced by sound quality [*F*_(1.74,41.71)_ = 1.16, *p* = 0.32, partial eta-squared = 0.05, 90% CI (0.00, 0.15)], or by action [*F*_(1,24)_ = 0.01, *p* = 0.91, $\;\eta {\;}_{\text{p}}^{2}$ < 0.001, 90% CI (0.00, 0.04)], or by critical sound quality–action interaction [*F*_(1.89,45.30)_ = 0.73, *p* = 0.48, $\;\eta {\;}_{\text{p}}^{2}$ = 0.03, 90% CI (0.00, 0.11)]. The very high percentage of correct responses suggests that the degradation did not affect understanding of the words.

**Table 1:** Auditory VAAST: accuracy of participants.

**Action**	**Sound Quality**	**Accuracy (*M* ±*SD*)**
Move backward	Undistorted	98.0 ± 4.6%
Move backward	Slightly distorted	98.0 ± 3.5%
Move backward	Moderately distorted	97.0 ± 6.0%
Move forward	Undistorted	97.4 ± 4.8%
Move forward	Slightly distorted	98.2 ± 4.3%
Move forward	Moderately distorted	97.8 ± 5.4%

#### A-VAAST: Reaction Times

**Figure 2:**
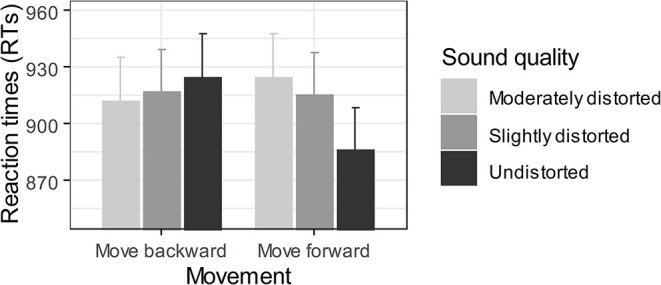
Averaged RTs (ms) as a function of sound quality and action. Vertical bars represent the standard error of the mean.

We first excluded incorrect trials (2.3%), as well as trials with RTs faster than 300 ms (no trials removed) and slower than 1,500 ms (5.0%). For the remaining correct trials, RTs falling outside 2.5 *SD* from the mean of each participant computed for each experimental condition level (action × sound quality) were also considered outliers and excluded from further analysis (0.9%). We then performed a repeated-measures ANOVA with the factors sound quality (undistorted, slightly distorted, and moderately distorted) and action (“move forward,” “move backward”) as within-subject factors for RTs of the remaining participants. Analyses neither revealed a significant main effect of the sound quality [*F*_(1.64,39.39)_ = 1.15, *p* = 0.32, $\;\eta {\;}_{\text{p}}^{2}$ = 0.05, 90% CI (0.00, 0.16)] nor of the type of action [*F*_(1,24)_ = 0.50, *p* = 0.49, $\;\eta {\;}_{\text{p}}^{2}$ = 0.02, 90% CI (0.00, 0.17)]. However, as expected, the critical interaction between the factors sound quality and action was significant [*F*_(1.79,43.05)_ = 5.88, *p* = 0.007, $\;\eta {\;}_{\text{p}}^{2}$ = 0.20, 90% CI (0.04, 0.34)] (Figure 2), suggesting that the effect of sound distortion on RTs depended on the action type of participants. To characterize this interaction, we performed two repeated-measures ANOVAs to evaluate the effect of sound quality on RTs separately for the two types of action (“move forward” and “move backward”). The sound quality effect was significant on the “move forward” [*F*_(1.79,43.01)_ = 7.02, *p* = 0.003, $\;\eta {\;}_{\text{p}}^{2}$ = 0.23, 90% CI (0.06, 0.37)] but not on the “move backward” [*F*_(1.64,39.33)_ = 0.45, *p* = 0.60, $\;\eta {\;}_{\text{p}}^{2}$ = 0.02, 90% CI (0.00, 0.10)] condition. Further, one-tailed Bonferroni corrected paired *t*-tests revealed that participants moved forward faster for undistorted words (886 ± 115 ms) than they did for either moderately distorted words (925 ± 117 ms) [*t*(24) = 4.32, *p* < 0.001, *d*_*z*_ = 0.86, 95% CI (0.40, 1.32)] or slightly distorted words (915 ± 113 ms) [*t*(24) = 2.69, *p* = 0.02, *d*_*z*_ = 0.54, 95% CI (0.11, 0.95)]. No difference was observed between moderately and slightly distorted words [*t*(24) = 0.77, *p* = 0.67, *d*_*z*_ = 0.15, 95% CI (−0.24, 0.55)].

### Discussion Experiment 1

In this experiment, participants were instructed to move forward and backward from a loudspeaker as quickly as possible, depending on the spoken word category (i.e., living being, inanimate object). Words were presented at different levels of distortion (i.e., undistorted, slightly distorted, and moderately distorted), which allowed us to measure the impact of sound quality on the approach/avoidance reactions of participants. As expected, we observed a critical interaction between sound quality and action on RTs of participants (i.e., the approach/avoidance compatibility effect). Further analysis revealed that sound quality only influenced forward (not backward) movements. In particular, undistorted words induced faster forward movements compared with those of moderately and slightly distorted words, suggesting that this effect was principally driven by undistorted words.

## Experiment 2

### Materials and Methods

#### Participants

A total of 26 panelists (18 females and eight males; French and/or Swiss citizens) recruited from all departments of Firmenich SA participated in this experiment. The average age of the panel was 45.1 years (*SD* = 10.7). It was conducted in accordance with the ethical principles stated in the Declaration of Helsinki issued by the World Medical Association. All participants provided written informed consent and were free to withdraw at any time without giving any reason. At the end of the panel, participants received snacks as a gesture of appreciation. To the best of our knowledge, this is the first study aiming to adapt the VAAST to olfactory stimuli. A priori determination of sample size was therefore not possible. Consequently, we conducted a sensitivity power analysis using G^*^Power 3 (Faul et al., 2007) to identify the smallest effect size that our sample would be able to detect in paired *t*-tests performed in RT analysis (section O-VAAST: Reaction times). The analysis revealed that our sample size (*n* = 26) could reliably detect effect sizes of Cohen's *d*_*z*_ = 0.50 (one-tailed), assuming α = 0.05 and power *(1 –* β*)* = 0.80.

#### Materials

##### Visual Scene

**Figure 3:**
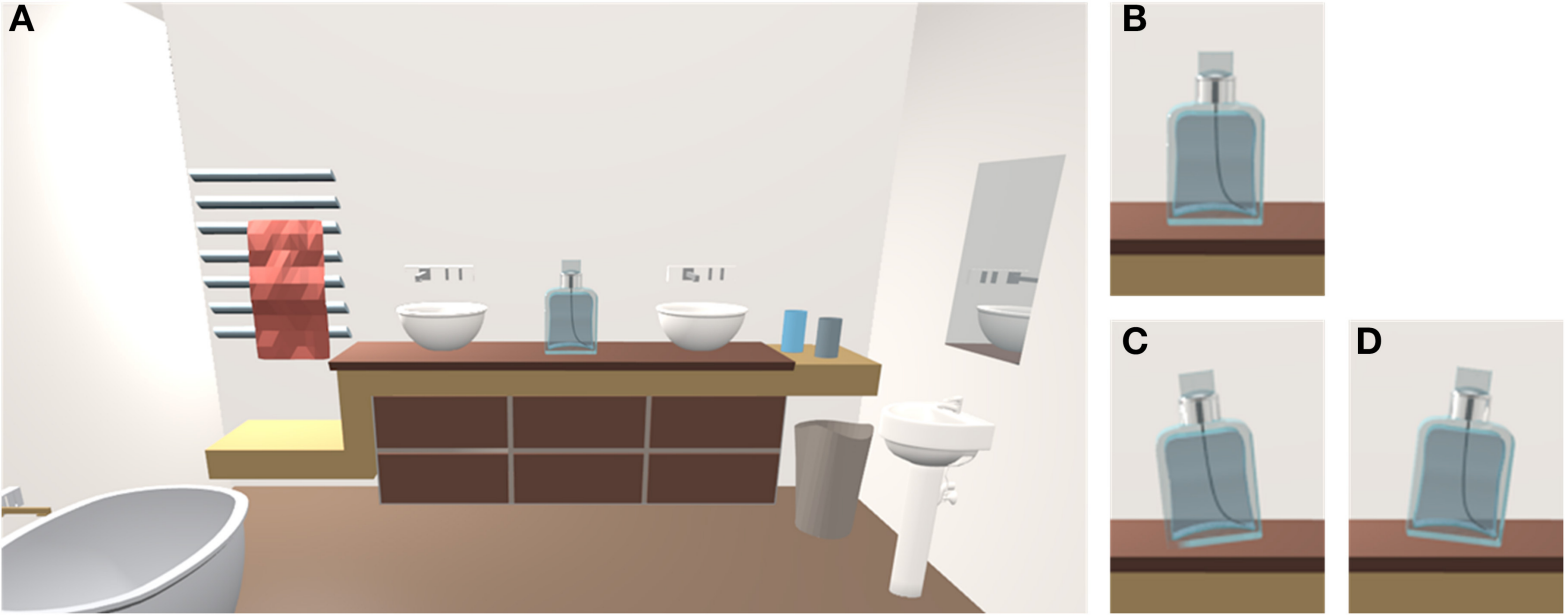
**(A)** Visual scene of the Olfactory Visual Approach/Avoidance by the Self Task (O-VAAST). **(B)** Perfume bottle in the vertical position (neutral stimulus). **(C,D)** Perfume bottle tilted to the left or right (visual targets of the task).

A virtual visual scene was designed for the Olfactory VAAST (O-VAAST) with 3D Blender. The visual scene consisted of a bathroom presented in the first-person view (Figure 3A). In the center of the visual scene, a shelf was displayed, above which was positioned a perfume bottle (i.e., the source of olfactory cues). The perfume bottle could be presented in the vertical position (neutral stimulus, Figure 3B) or tilted to the left or right (visual targets of the O-VAAST; Figures 3C,D, respectively).

##### Olfactory Stimuli

We performed a preliminary study to select the odorants for the O-VAAST and identify suitable concentrations. Using visual analog scales (ranging from 0 to 100), 26 internal panelists (mean age = 41.9, *SD* = 12.6 years; 17 females and nine males; French and/or Swiss citizens) were asked to evaluate the perceived pleasantness (from “very unpleasant” to “very pleasant”), intensity (from “not perceived” to “very strong”), and familiarity (from “not familiar at all” to “very familiar”) of pleasant (Perfume 1, Perfume 2) and unpleasant [butyric acid, synthetic body odor (SBO)] odors diluted at different concentrations in dipropylene glycol (DIPG) or isopropyl myristate (IPM). Perfume 1 was a commercial deodorant for women described by Firmenich perfumers as “floral,” “green,” “geranium,” “citrus,” and “aromatic.” Perfume 2 was a commercial deodorant for men described by Firmenich perfumers as “floral,” “fruity,” “citrus,” “woody,” “musky,” and “ambery.” From the results (see Table 2), we selected two odors that maximized differences in liking and minimized differences in both intensity and familiarity. Perfume 1 diluted at 10% in DIPG (presented below as “Perfume”) was then selected as the pleasant odor, while SBO diluted at 1% in IPM (presented below as “SBO”) was selected as the unpleasant odor. In the O-VAAST, DIPG was also used as the neutral odorless condition.

**Table 2:** Pretested set of odors.

**Product**	**Solvent**	**Liking (*M* ±*SD*)**	**Intensity (*M* ±*SD*)**	**Familiarity (*M* ±*SD*)**
Perfume 1, 1%	DIPG	52.2 ± 23.4	54.4 ± 17.8	59.4 ± 16.2
Perfume 1, 10%	DIPG	73.3 ± 18.3	66.4 ± 16.1	67.9 ± 15.9
Perfume 2, 1%	DIPG	46.2 ± 23.5	53.5 ± 20.3	58.7 ± 17.9
Perfume 2, 10%	DIPG	67.1 ± 20.4	68.2 ± 15.2	70.2 ± 16.7
Butyric acid, 0.5%	DIPG	7.1 ± 9.2	75.0 ± 18.4	72.7 ± 16.9
Butyric acid, 1%	DIPG	7.6 ± 12.3	81.9 ± 15.9	73.5 ± 18.6
SBO 0.001%	IPM	46.6 ± 18.8	36.5 ± 20.6	55.6 ± 20.5
SBO 0.005%	IPM	47.0 ± 16.6	32.0 ± 23.9	50.2 ± 17.7
SBO 0.1%	IPM	33.8 ± 22.2	51.2 ± 94.1	60.7 ± 21.8
SBO 1%	IPM	19.3 ± 21.7	62.9 ± 19.6	74.9 ± 18.0

##### Olfactory Display System

The selected odors were then placed inside three glass vials and arranged in a custom-built computer-controlled olfactory display system (see Ischer et al., 2014). During the interstimulus interval, air valves were opened, thus delivering clean air to the nose of participants. During the delivery of odor, air valves were automatically closed and odor valves opened. As typically performed in our implicit olfactory procedures (Lemercier-Talbot et al., 2019; Cereghetti et al., 2020), the interstimulus interval and the odorant flow rate were both fixed at 2 L × min^−1^, thus delivering a constant flow to the nose of participants. Stainless steel tips were used as the final delivery piece.

#### Procedure

The procedure consisted of two successive phases: the odor evaluation phase and the O-VAAST.

##### Odor Evaluation Phase

In the odor evaluation phase, participants were instructed that they would be provided with odors to evaluate. The odors were administered in a random order for 6 s. After each olfactory stimulation, participants assessed pleasantness (from “very unpleasant” to “very pleasant”), intensity (from “not perceived” to “very strong”), and familiarity (from “not familiar at all” to “very familiar”) by using visual analogic scales that ranged from 0 to 100 (see Delplanque et al., 2008, for more details).

##### O-VAAST

Developed in the MATLAB environment (ver. R2014b, The MathWorks, Inc., Natick, Massachusetts, United States) using the Psychtoolbox library (version 3.0.11), the O-VAAST consisted of two successive phases, namely, practice and test. In the practice phase, participants were told that they would be immersed in a virtual environment, i.e., a bathroom equipped with a perfume bottle. Participants were asked to move forward or backward from the perfume bottle as quickly and accurately as possible, depending on its inclination (i.e., left or right). The correspondence between the perfume bottle inclination and the approach/avoidance action was counterbalanced across participants: Half of the participants had to approach the left-tilted perfume bottles and avoid the right-tilted ones, whereas the remaining half had to approach the right-tilted perfume bottles and avoid the left-tilted ones.

In practice trials, the visual scene was presented full screen with the perfume bottle tilted to the left or right (visual target). Depending on its inclination, participants had to press key 8 of the numeric keypad three times successively to approach the perfume bottle and key 2 of the numeric keypad to move away from it. After each correct key-press response, the whole visual scene was zoomed in or out by 10%, giving the visual impression of walking forward or backward as a consequence of the approach/avoidance action of participants (Rougier et al., 2018). Following a wrong response, a red capital “X” was displayed for 500 ms and participants were required to provide the correct response to trigger the visual flow. Successive trials were separated by a fixation cross presented in the center of a blank screen for 250 ms. The practice phase was composed of four “move forward” and four “move backward” trials presented in a randomized order.

The test phase was similar, and the difference is that three olfactory stimuli (Perfume, SBO, and DIPG) were delivered as primes prior to the approach/avoidance actions of participants. In that respect, participants were informed that they would smell the odors coming from the perfume bottle throughout the task. The testing phase consisted of 24 odor blocks. In each block, the delivery of an olfactory cue was followed by four visual targets, i.e., four tilted perfume bottles presented consecutively (T1, T2, T3, and T4). At the beginning of each block, the visual scene was presented full screen with the perfume bottle in the vertical position (i.e., the neutral visual scene); participants were then instructed to press the “start” key (key 5 of the numeric keypad). This action triggered a 3-s countdown timer (3, 2, 1) displayed in the center of the vertical perfume bottle, after which an asterisk (^*^) was presented as the sniffing signal for 2 s. The odor was then delivered, and participants were instructed to inhale at this moment. In reality, the odor valve was opened 1 s before the onset of the sniffing signal, ensuring the presence of the odorant in the final delivery pieces of the olfactometer during participants' inhalation. The offset of the sniffing signal matched the closing of the odorant valve, which was thus opened for a total of 3 s. After olfactory priming, the vertical perfume bottle was replaced by T1. Participants thus had to approach/avoid the perfume bottle by following the inclination-action rules that they trained for during the practice phase. The correct response was then followed by a fixation cross presented in the center of a blank screen for 250 ms, after which the neutral visual scene was presented again. Participants thus had to again press the “start” key, this time to trigger the next trial within the block. A fixation cross was then presented in the center of the vertical perfume bottle for 500 ms, after which T2 was presented. This sequence also applied to T3 and T4; the difference is that after the response of participants to T4, the duration of the blank screen was increased from 250 ms to 4 s to prevent odor adaptation and habituation. Although participants had to press the relevant key three times consecutively to approach the perfume bottle or to move away from it, we considered accuracy and RT only for the first action of participants (see Rougier et al., 2018).

Across the 24 blocks, each participant was exposed to the perfume eight times, to the SBO eight times, and to the DIPG eight times, and was asked to respond in total to 96 tilted perfume bottles (48 “move forward” and 48 “move backward” trials). The order of presentation of odor and perfume bottle inclination was randomized for each participant. In particular, randomization of perfume bottle inclination was controlled at an individual level, ensuring that each inclination (left-tilted, right-tilted) was presented four times for each olfactory cue (Perfume, SBO, DIPG) × trial (T1, T2, T3, T4) level. Within a block, the four perfume bottles could be tilted to the same or to different directions, making it impossible to anticipate T3 and/or T4 responses from previous responses.

### Results

Experimental data and analysis scripts are available on the OpenScience Framework platform at https://osf.io/jkr7m/

#### Odor Evaluation Phase

**Figure 4:**
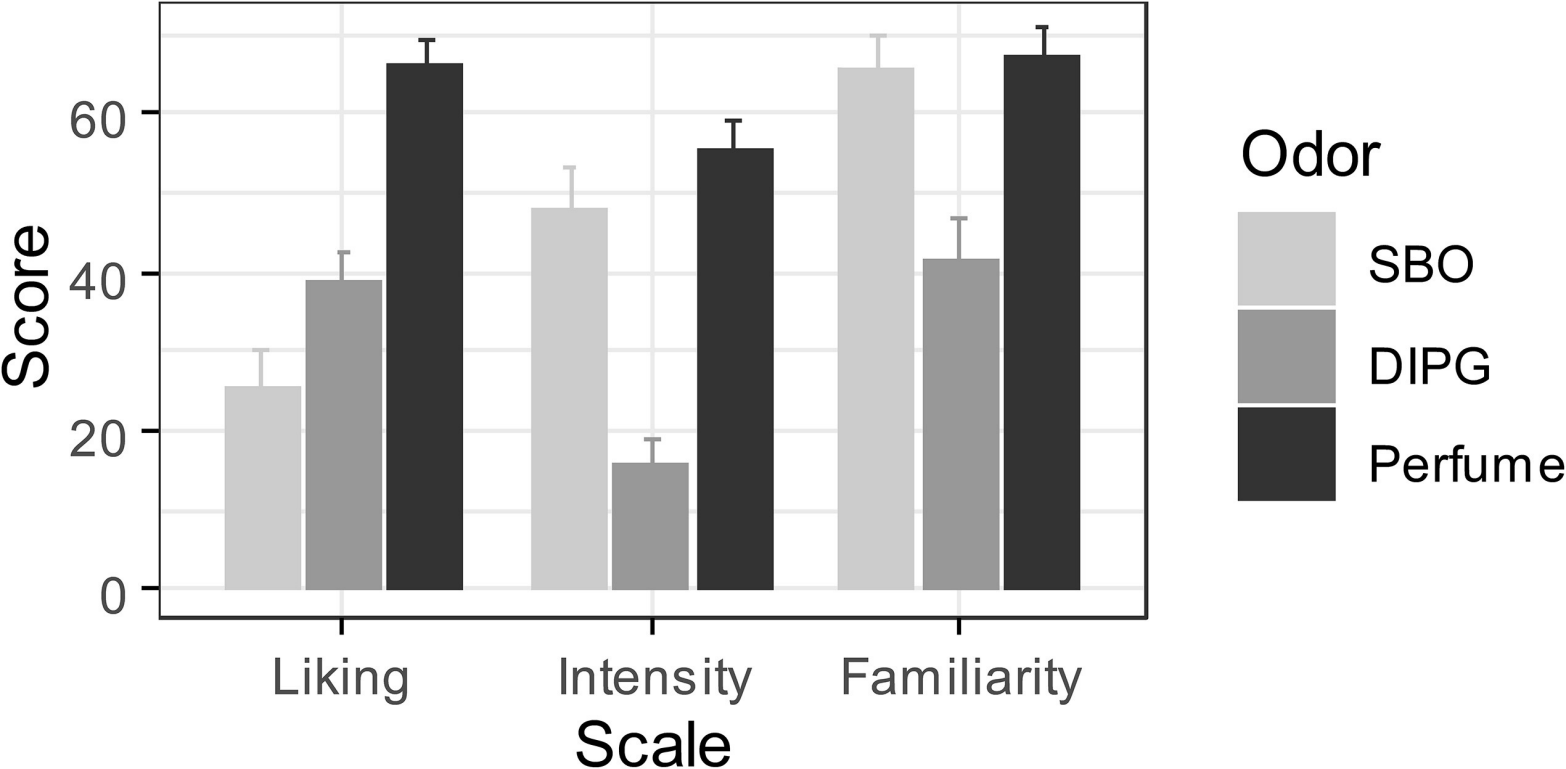
Results on liking, intensity, and familiarity scales on Perfume, SBO, and DIPG.

Three repeated-measures ANOVAs were performed to evaluate the differences in perceived intensity, familiarity, and liking between the three odors. A significant effect of odor was observed on perceived liking [*F*_(1.95,48.83)_ = 36.03, *p* < 0.0001, $\;\eta {\;}_{\text{p}}^{2}$ = 0.59, 90% CI (0.42, 0.68)], intensity [*F*_(1.93,48.32)_ = 37.10, *p* < 0.0001, $\;\eta {\;}_{\text{p}}^{2}$ = 0.60, 90% CI (0.43, 0.68)], and familiarity [*F*_(1.84,46.05)_ = 12.86, *p* < 0.0001, $\;\eta {\;}_{\text{p}}^{2}$ = 0.34, 90% CI (0.15, 0.47)] (Figure 4). Two-by-two comparisons were then conducted by using a series of paired *t*-tests with Bonferroni correction. Not surprisingly, Perfume was perceived as being more pleasant than DIPG [*t*(25) = 6.05, *p* ≤ 0.0001, *d*_*z*_ = 1.19, 95% CI (0.67, 1.68)] and SBO [*t*(25) = 8.20, *p* ≤ 0.0001, *d*_*z*_ = 1.61, 95% CI (1.02, 2.19)]. As expected, the SBO was also rated as being less pleasant than the DIPG [*t*(25) = −2.60, *p* = 0.047, *d*_*z*_ = −0.51, 95% CI (−0.91, −0.10)]. The two odorants were also perceived as being more intense [Perfume: *t*(25) = 7.98, *p* ≤ 0.0001, *d*_*z*_ = 1.57, 95% CI (0.98, 2.14); SBO: *t*(25) = 7.20, *p* ≤ 0.0001, *d*_*z*_ = 1.41, 95 < % CI (0.86, 1.95)] and more familiar [Perfume: *t*(25) = 4.18, *p* < 0.001, *d*_*z*_ = 0.82, 95% CI (0.37, 1.26); SBO: *t*(25) = 4.02, *p* < 0.001, *d*_*z*_ = 0.79, 95% CI (0.34, 1.22)] than the DIPG. In contrast, we did not find any significant difference between Perfume and SBO in intensity [*t*(25) = 1.49, *p* = 0.44, *d*_*z*_ = 0.29, 95% CI (−0.10, 0.68)] or familiarity [*t*(25) = 0.37, *p* = 0.71, *d*_*z*_ = 0.07, 95% CI (−0.31, 0.46)]. Critically, these results allowed us to exclude that a potential impact of the odors in the O-VAAST, analyzed below, could be attributed to differences in intensity or familiarity (i.e., differences other than valence).

#### O-VAAST: Accuracy

Accuracy of participants on the O-VAAST across the six conditions is summarized in Table 3. We performed a repeated-measures ANOVA with the factors olfactory cue (Perfume, SBO, DIPG) and action (“move forward,” “move backward”) as within-subject factors for the response accuracy of participants. A significant effect of action [*F*(1, 25) = 4.67, *p* = 0.04, $\;\eta {\;}_{\text{p}}^{2}$ = 0.16, 90% CI (0.00, 0.36)] reflected better accuracy for “move backward” trials (*M* = 97.8%, *SD* = 2.9%) than for “move forward” trials (*M* = 96.6%, *SD* = 3.1%). Nevertheless, accuracy was influenced by neither the olfactory cue [*F*_(1.91,47.84)_ = 0.88, *p* = 0.42, $\;\eta {\;}_{\text{p}}^{2}$ = 0.03, 90% CI (0.00, 0.12)] nor the cue–action interaction [*F*_(1.95,48.76)_ = 1.29, *p* = 0.28, $\;\eta {\;}_{\text{p}}^{2}$ = 0.05, 90% CI (0.00, 0.15)].

**Table 3:** Olfactory VAAST: accuracy of participants.

**Action**	**Olfactory cue**	**Accuracy (*M* ±*SD*)**
Move backward	DIPG	97.8 ± 3.9%
Move backward	Perfume	97.6 ± 4.4%
Move backward	SBO	98.1 ± 3.4%
Move forward	DIPG	95.4 ± 6.3%
Move forward	Perfume	97.8 ± 3.9%
Move forward	SBO	96.6 ± 5.1%

#### O-VAAST: Reaction Times

**Figure 5:**
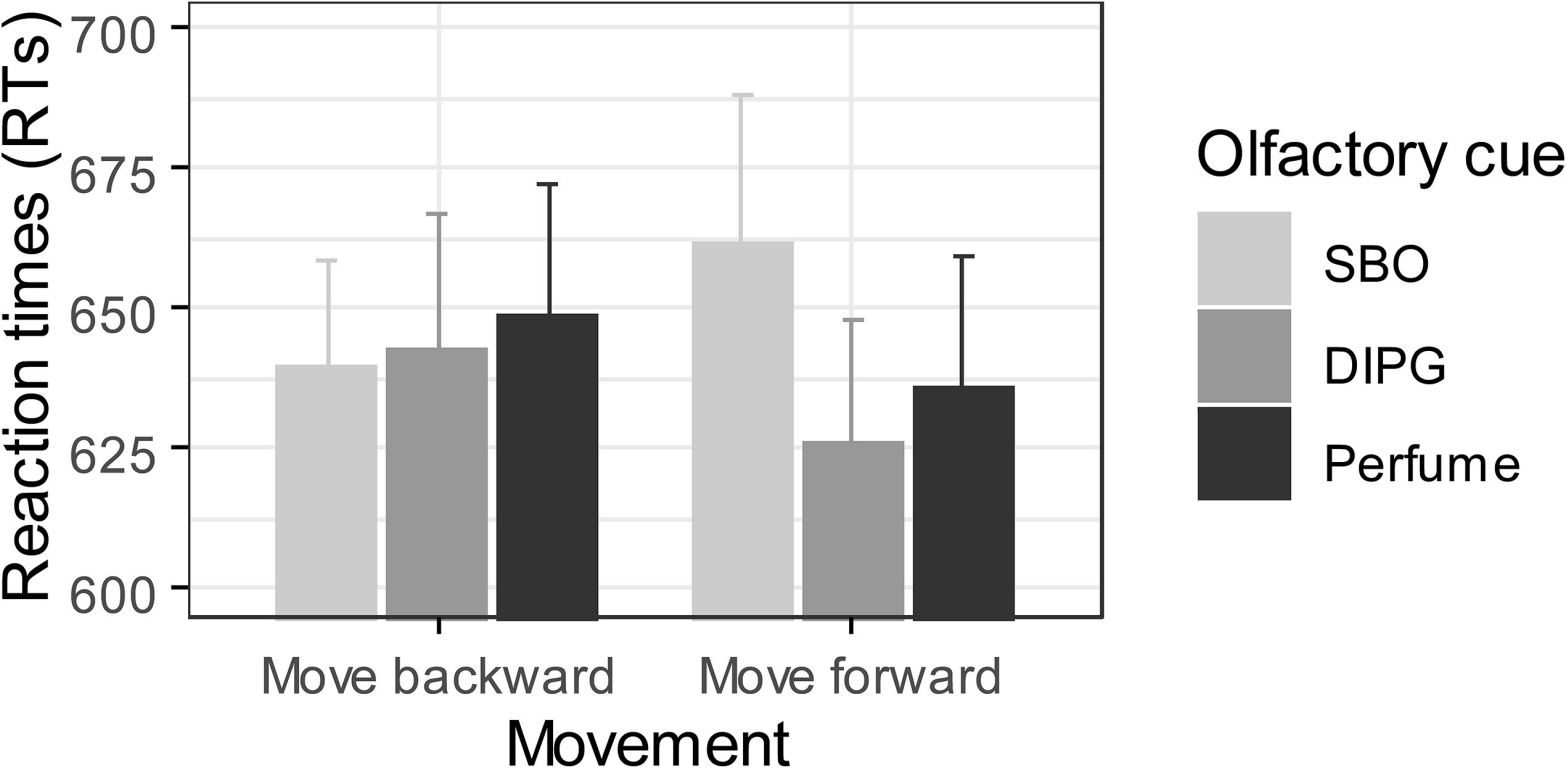
Test phase: averaged RTs (ms) as a function of olfactory cue and action. Vertical bars represent the standard error of the mean.

Similar to the previous study, we first excluded incorrect trials (2.8%), as well as trials with RTs faster than 300 ms (1.1%) and slower than 1,500 ms (2.2%). For the remaining correct trials, RTs falling outside 2.5 *SD* from the mean of each participant computed at each target position level (T1, T2, T3, and T4) were also considered outliers and excluded from further analysis (2.2%). We then performed a repeated-measures ANOVA with the factors olfactory cue (Perfume, SBO, DIPG) and action (“move forward,” “move backward”) as within-subject factors for valid RTs. Analyses neither revealed a significant main effect of the olfactory cue [*F*_(1.95,48.87)_ = 1.62, *p* = 0.21, $\;\eta {\;}_{\text{p}}^{2}$ = 0.06, 90% CI (0.00, 0.17)] nor of the type of action [*F*_(1,25)_ = 0.06, *p* = 0.81, $\;\eta {\;}_{\text{p}}^{2}$ = 0.002, 90% CI (0.00, 0.09)]. However, notably, we found a critical significant interaction between action and olfactory cue [*F*_(1.84,46.10)_ = 3.80, *p* = 0.03, $\;\eta {\;}_{\text{p}}^{2}$ = 0.13, 90% CI [0.01, 0.26)] (Figure 5), suggesting that the effect of odor pleasantness on RTs depended on the action type of participants. To characterize this interaction, we performed two repeated-measures ANOVAs to evaluate the cue effect (Perfume, SBO, DIPG) across the two move actions (“move forward” and “move backward”). The cue effect was significant on the “move forward” [*F*_(1.93,48.22)_ = 5.22, *p* = 0. 01, $\;\eta {\;}_{\text{p}}^{2}$ = 0.17, 90% CI (0.03, 0.30)] but not on the “move backward” [*F*_(1.98,49.60)_ = 0.31, *p* = 0.73, $\;\eta {\;}_{\text{p}}^{2}$ = 0.01, 90% CI (0.00, 0.07)] condition. Further, one-tailed Bonferroni-corrected paired *t*-tests revealed that participants moved forward more slowly after being primed with the SBO (*M* = 661, *SD* = 136 ms) than after being primed by either the DIPG (*M* = 626, *SD* = 111 ms) [*t*(25) = 2.86, *p* = 0.01, *d*_*z*_ = 0.56, 95% CI (0.14, 0.97)] or the Perfume (*M* = 636, *SD* = 119 ms) [*t*(25) = 2.42, *p* = 0.03, *d*_*z*_ = 0.47, 95% CI (0.06, 0.88)]. No difference was observed between DIPG and Perfume [*t*(25) = 0.87, *p* = 1.00, *d*_*z*_ = 0.17, 95% CI (−0.22, 0.56)].

### Discussion Experiment 2

In this experiment, participants were asked to move forward or backward from the perfume bottle as quickly as possible, depending on its inclination (i.e., left or right). Three olfactory stimuli (Perfume, SBO, and DIPG) were delivered as primes, which allowed us to measure the impact of odor pleasantness on approach/avoidance reactions of participants. As expected, we observed a critical interaction between odor pleasantness and action on RTs of participants (i.e., the approach/avoidance compatibility effect). Further analysis revealed that odor pleasantness only influenced forward (not backward) movements. In particular, unpleasant odors (SBO) induced slower forward movements compared with those of neutral-to-pleasant odors (DIPG, Perfume), suggesting that this effect was principally driven by unpleasant odors.

## General Discussion

The main purpose of the present study was to extend the VAAST (Rougier et al., 2018) to the auditory and olfactory modalities. More specifically, we aimed to (1) validate new procedures to measure the ability of sounds and odors to elicit automatic approach/reactions toward their relative source, and (2) provide further evidence concerning the ability of VAAST to discriminate emotional stimuli that differ in their hedonic value, using approach/avoidance compatibility effects as implicit measures of hedonicity. In Experiment 1, participants were asked to move forward or backward from a loudspeaker that emitted spoken words presented at different levels of distortion (i.e., undistorted, slightly distorted, and moderately distorted); our assumption is that the more distorted the signal, the more unpleasant the stimulus. In Experiment 2, participants were asked to move forward or backward from a perfume bottle that delivered pleasant and unpleasant odors. We predicted that, consistent with the approach/avoidance compatibility effect, faster RTs would be required to approach positive stimuli and to avoid negative stimuli than was the case for the reverse. In both experiments, we observed an expected interaction between the direction of the movement induced by the responses of participants (forward or backward) and the quality of the emotional stimulus. More precisely, undistorted words and neutral-to-pleasant odors (DIPG, Perfume) induced faster forward movements toward their source than did (moderately and slightly) distorted words and unpleasant odors (SBO). However, although the direction of the results was congruent with our hypothesis, the quality of the stimulation did not impact the backward movements of participants. This asymmetry of the compatibility effect could be related to the nature of the stimuli used in our experiments: Neither the emotional stimuli (auditory and olfactory cues) nor the visual scenes (including sources and the surrounding environments) were likely to be appraised as dangerous events that may induce fear and elicit avoidance reactions. That is, the methodology presented here is not intended to be used with strong emotional stimuli, especially negative stimuli, but rather with positive stimuli that slightly differ in their hedonic value. Consequently, to further develop the method, one could consider (1) dropping the avoidance condition and (2) contrasting the approach movement to a condition without movement.

Notably, our results confirmed that the VAAST is able to measure compatibility effects with a relatively small sample size, providing further evidence of its robustness and validity. According to Rougier et al. (2018), 35 subjects are enough to identify compatibility effects in the VAAST. Moreover, since (1) we used stronger emotional stimuli (in particular odors) and since (2) we implemented a behavioral task with higher ecological validity, we expected our tasks to be more sensitive. Indeed, the effect sizes observed in significant *t*-tests performed in RT analysis (Experiment 1: *d*_z_ = 0.86 and *d*_*z*_ = 0.54; Experiment 2: *d*_z_ = 0.56 and *d*_z_ = 0.47) were higher, or quite close, to the minimum effect size that can be detected with our sample size (Experiment 1: *d*_z_ = 0.51; Experiment 2: *d*_z_ = 0.50), assuming α = 0.05 and power *(1 –* β*)* = 0.80.

Most studies conducted so far on the VAAST used explicit instructions (i.e., procedures in which participants are explicitly asked to process the valence of the emotional stimulus, see Rougier et al., 2018). Importantly, our study also supported the evidence that relatively large approach (but not avoidance) compatibility effects ($\eta_{p}^{2}$ = 0.20 in Experiment 1; $\eta_{p}^{2}$ = 0.13 in Experiment 2) can be observed when using implicit instructions, i.e., without requiring participants to explicitly attend the valence of the emotional stimulus (Phaf et al., 2014). In Experiment 1, participants were instructed to respond to the word category, regardless of the level of distortion at which these stimuli were presented. That is, they were required to explicitly process a feature of emotional stimuli other than valence. In Experiment 2, participants were instructed to respond to the perfume bottle inclination, regardless of the olfactory cues delivered by the olfactometer. Experiment 2 thus went further by demonstrating that compatibility effects can be found without requiring participants to explicitly process the stimuli that induce emotion, the latter being task irrelevant. In that sense, the VAAST may be a privileged procedure to measure approach/avoidance reactions by using implicit instructions, especially when multiple sensory modalities are involved. In this case, target stimuli and emotional stimuli could be presented in different sensory modalities (as in Experiment 2), thus providing the best conditions for applying implicit instructions. Moreover, the VAAST appears to be, by its nature, a procedure that fits particularly well in cross-modal research: One could imagine its use with very diverse emotional stimuli, including visual (as in the original version of the VAAST), auditory, and olfactory stimuli (as in the present study), but potentially also gustatory and haptic/tactile stimuli. In this case, the challenge for the investigator will be to identify a visual context (toward which the approach/avoidance reactions are directed) that is meaningfully linked to the emotional stimuli.

The choice of the task is also crucial because it defines the cognitive processes involved during its completion, potentially affecting the sensitivity of the measure, i.e., the approach/avoidance compatibility effect sizes. In the present study, RTs were higher in Experiment 1 (*M* = 913, *SD* = 104 ms) than in Experiment 2 (*M* = 643, *SD* = 111 ms). This difference is mostly due to the nature of cognitive processes involved in the two tasks. In Experiment 1, the task required semantic processing of spoken words. This was not the case in the Experiment 2, in which participants had to discriminate an elementary feature of visual targets (i.e., their inclination). Furthermore, as shown in different studies based on a dual-task paradigm (Gagné et al., 2017) and on cognitively overloaded auditory tasks (Gros et al., 2008), audio degradation could induce an increase in listening effort, resulting in an increase in RTs. However, in that case, lengthening of RTs would appear for both backward and forward movements, which was not the case in our experiment. We can thus exclude the possibility that the levels of degradation used in our (non-cognitively overloaded) auditory VAAST induced an increase in listening effort. Obviously, if we had used higher levels of degradation, differences in RTs would not specifically reflect the hedonicity of the listening experience, but rather the difficulty of understanding, or perhaps the intelligibility loss. In this case, either a lengthening of RTs would appear for highly distorted sounds regardless of the movement, or faster approach movements would be observed for highly distorted sounds only, with participants getting closer to the loudspeaker to better understand them. An experiment is in progress with our team to verify the outcome.

The VAAST is likely to have higher ecological validity compared with that of explicit measures, and even compared with that of other implicit measures. First, the VAAST captures cognitive processes that “naturally” occur when emotional stimuli are encountered. In the so-called ecological situations, that is in “real-life” contexts, individuals rarely express their perceptual judgments explicitly. Perceptual feelings are nevertheless likely to influence decisions and behaviors of individuals in their daily life. On the basis of the idea that approach/avoidance behaviors belong to a critical adaptive process (Tooby and Cosmides, 1990), Bargh (1997) argues that encountered stimuli are automatically evaluated by the cognitive system on a positive/negative dimension. This evaluation leads to the activation of behavioral predispositions (Chen and Bargh, 1999) that finally elicit approach/avoidance reactions. These predispositions, which are activated in ecological situations, are also activated during the VAAST, making it a procedure able to capture cognitive processes occurring in “real-life” contexts. Second, by providing visual feedback mimicking whole-body movements in relation to the emotional stimulus, the VAAST clearly simulates what happens in “real life” when such a stimulus is approached or avoided. Importantly, although in the original VAAST (Rougier et al., 2018) participants had to approach/avoid words located in a non-congruent environment (a corridor in Experiments 1, 2, 3, and 5; a street in Experiments 4 and 6), in our procedures, participants had to approach/avoid 3D virtual objects (i.e., a loudspeaker and a perfume bottle) located in a congruent environment (i.e., a living room and a bathroom), thus increasing the ecological validity of our procedures. We hope that future research will further explore the potential of the VAAST to measure behavioral tendencies in ecological situations by using 3D virtual objects as target stimuli instead of emotional words.

The VAAST could be a very promising procedure to capture cognitive processes that are “naturally” involved during product experience. Indeed, one could argue that the perception of a product (e.g., loudspeaker, perfume), its related cues (e.g., auditory, olfactory), and its conditioned stimuli (e.g., brand, packaging) could lead to the activation of behavioral tendencies that promote product purchase or consumption. The visual scene, including sources (e.g., loudspeaker, perfume bottle) and the surrounding environments (e.g., living room, bathroom), can potentially be manipulated by the investigator to test the effect of specific contextual factors on reactions of consumers toward products, thus making the VAAST a contextual-rich procedure that, we are certain, has much potential in consumer research.

## Conclusion

Here, we presented an adapted version of the VAAST (Rougier et al., 2018) that allowed us to measure the ability of auditory and olfactory cues to trigger automatic approach/avoidance reactions toward their source. In two experiments, we showed that the VAAST can successfully be adapted to modalities other than visual.

## Data Availability Statement

The original contributions presented in the study are publicly available. This data can be found here: https://osf.io/jkr7m/.

## Ethics Statement

Ethical review and approval was not required for the study on human participants in accordance with the local legislation and institutional requirements. The patients/participants provided their written informed consent to participate in this study.

## Author Contributions

DC, PF, LG, LM, ED, IC, and RV designed the studies. LM implemented the A-VAAST. DC implemented the O-VAAST. LM ran the first experiment. DC and LM ran the second experiment. DC and LM analyzed and interpreted the data of both studies. DC, TH, and RV wrote the manuscript. All authors contributed to the article and approved the submitted version.

## Conflict of Interest

DC and IC were employed by Firmenich SA. LG was employed by Orange Labs. ED was employed by PSA Groupe. TH was employed by Silliker SAS, Mérieux NutriSciences. The remaining authors declare that the research was conducted in the absence of any commercial or financial relationships that could be construed as a potential conflict of interest.

## Publisher's Note

All claims expressed in this article are solely those of the authors and do not necessarily represent those of their affiliated organizations, or those of the publisher, the editors and the reviewers. Any product that may be evaluated in this article, or claim that may be made by its manufacturer, is not guaranteed or endorsed by the publisher.
